# A Novel Anti-CD47 Nanobody Tetramer for Cancer Therapy

**DOI:** 10.3390/antib13010002

**Published:** 2024-01-02

**Authors:** Nataliya M. Ratnikova, Yulia Kravchenko, Anna Ivanova, Vladislav Zhuchkov, Elena Frolova, Stepan Chumakov

**Affiliations:** 1 Shemyakin-Ovchinnikov Institute of Bioorganic Chemistry, Russian Academy of Sciences, Moscow 117997, Russia; n89ratnikova@gmail.com (N.M.R.); vladik55318@gmail.com (V.Z.);; 2 Winogradsky Institute of Microbiology, FRC Biotechnology Russian Academy of Sciences, Moscow 119071, Russia; 3 Kulakov National Medical Research Center of Obstetrics, Gynecology and Perinatology, Moscow 117997, Russia

**Keywords:** CD47, SIRPa, nanobody, VHH, streptabody, immunotherapy, phagocytosis

## Abstract

CD47 acts as a defense mechanism for tumor cells by sending a “don’t eat me” signal via its bond with SIRPα. With CD47’s overexpression linked to poor cancer outcomes, its pathway has become a target in cancer immunotherapy. Though monoclonal antibodies offer specificity, they have limitations like the large size and production costs. Nanobodies, due to their small size and unique properties, present a promising therapeutic alternative. In our study, a high-affinity anti-CD47 nanobody was engineered from an immunized alpaca. We isolated a specific VHH from the phage library, which has nanomolar affinity to SIRPα, and constructed a streptavidin-based tetramer. The efficacy of the nanobody and its derivative was evaluated using various assays. The new nanobody demonstrated higher affinity than the monoclonal anti-CD47 antibody, B6H12.2. The nanobody and its derivatives also stimulated substantial phagocytosis of tumor cell lines and induced apoptosis in U937 cells, a response confirmed in both in vitro and in vivo settings. Our results underscore the potential of the engineered anti-CD47 nanobody as a promising candidate for cancer immunotherapy. The derived nanobody could offer a more effective, cost-efficient alternative to conventional antibodies in disrupting the CD47–SIRPα axis, opening doors for its standalone or combinatorial therapeutic applications in oncology.

## 1. Introduction

CD47, a cell surface glycoprotein belonging to the immunoglobulin superfamily, is ubiquitously expressed throughout the human body and participates in numerous cellular processes [1,2]. Within the immune system, CD47 functions as an innate checkpoint receptor [3]. Its interaction with one of its natural ligands, SIRPα (signal regulatory protein-alpha), which is expressed on the macrophage membrane, generates an anti-phagocytic “don’t eat me” signal [4,5,6].

CD47 overexpression is observed in various types of cancers, and this upregulation is considered a significant mechanism for evading innate immunity surveillance [4,5,7,8]. CD47 upregulation by tumors has been shown to correlate with poor outcomes of disease [9,10,11]. As a result, disrupting the CD47–SIRPα axis has emerged as a promising target in cancer immunotherapy, with extensive ongoing research.

Inhibition of the CD47–SIRPα interaction has stimulated broad-ranging antitumor T-cell immune responses in an array of preclinical models and human tumors in mouse xenotransplantation models [12,13,14,15]. At present, multiple humanized CD47-blocking antibodies are in clinical trials, either as standalone therapeutic agents or combined with additional drugs for the treatment of both solid and hematologic malignancies (Clinical Trials: NCT03763149, NCT02216409, NCT03013218, etc.).

Monoclonal antibodies offer high specificity and affinity to antigens, making them promising candidates for clinical applications. However, their large size (around 150 kDa), potential for immunogenicity, and considerable production costs may limit their practical use in clinical settings [16,17]. In response to these challenges, a new type of antibody, known as VHH antibodies or nanobodies, has gained prominence as a promising avenue for developing innovative therapeutic and diagnostic strategies. Nanobodies are single-variable domains (VHH, variable heavy–heavy, approximately 15 kDa) derived from the heavy-chain-only IgG antibodies (HcAbs) of the Camelidae family, making them the smallest known antigen-binding immunoglobulin fragments that retain full functionality [16]. They offer substantial benefits for tumor therapy, including their small size, high affinity, stability, and solubility. They are also relatively simple to produce in bacteria, yeast, or eukaryotic systems, with minimal associated costs; while this does not currently impact the pricing, it holds potential for enhancing future availability [18,19]. The compact size and unique paratope architecture of nanobodies, coupled with their unusually long third complementarity determining region (CDR3), enable them to recognize highly complex or hidden epitopes often inaccessible to conventional antibodies [20,21]. Nanobodies can penetrate the tumor milieu more effectively compared to full-sized monoclonal antibodies; however, their monomeric nature often leads to faster dissociation rates, reflecting a balance between size and binding kinetics. Furthermore, VHHs share sequence similarities with human type 3 VH regions, contributing to their low immunogenicity [22]. This property, in combination with the potential for further humanization of nanobodies, minimizes the risk of adverse immune responses [21,23]. The single-domain structure of VHHs and their lack of post-translational modifications [24] render nanobodies an ideal candidate for selection via phage display techniques. This feature also facilitates the generation of modified and complexed therapeutic agents, such as bispecific agents and tetravalent antibodies, further expanding their potential applications in cancer therapeutics.

In this study, we engineered a high-affinity anti-human CD47 nanobody from an immunized alpaca, demonstrating its effective disruption of the CD47–SIRPα interaction. A specific VHH was isolated from a constructed phage library, demonstrating nanomolar affinity to the immobilized natural ligand, CD47, as determined via surface plasmon resonance assays. This generated nanobody effectively displaced SIRPα binding to CD47 in a competitive ELISA, showing higher potency than the commercially available monoclonal anti-CD47 antibody, B6H12.2. Utilizing this selected anti-CD47 nanobody, we created a biotinylated derivative and a tetravalent molecule, referred to as a streptabody. These multimers exhibited enhanced affinity compared to the native anti-CD47 nanobodies. Both the nanobody and its derivatives stimulated substantial phagocytosis of MCF7 and U937 tumor cell lines via the primary macrophages ex vivo, thus validating the affinity and functional activity of the derived anti-CD47 nanobody. Moreover, this nanobody induced apoptosis in U937 cells, a finding corroborated by both in vitro studies and in vivo experiments involving murine xenotransplant models. Given these promising results, the resulting nanobody could be a valuable candidate for further in vivo evaluation, both as a standalone therapeutic agent and in combination with other drugs, in the field of cancer immunotherapy.

## 2. Materials and Methods

### 2.1. Reagents and Cell Lines

The MCF7 breast cancer cell line, HEK-293 and HEK-293T embryonic kidney cell lines, and monocytic lymphoma cell line U937 were purchased from ATCC^®^ (Manassas, Virginia, USA). The mouse anti-CD47 mAb B6H12 was either produced and purified from hybridoma HB9771 purchased from ATCC^®^, or purchased from Santa-Cruz Biotechnology (Dallas, TX, USA). The biotinylated mouse anti-CD47 mAb (clone B6H12.2) was purchased from BioLegend (San Diego, CA, USA). The monoclonal sheep anti-SIRPa antibodies and streptavidin-HRP were purchased from R&D Systems (Minneapolis, MN, USA). The monoclonal anti-M13-HRP antibody was from Thermo (Waltham, MA, USA). All the secondary antibodies were purchased from Santa-Cruz Biotechnology. The recombinant human SIRPa-Fc was from R&D Systems. The human CD47 with His tag was a kind gift from P.M. Chumakov. The streptavidin was purchased from MyBioSource (San Diego, CA, USA). The fluorescent lipophilic dyes DiO and DiL were from Biotium (San Francisco Bay Area, CA, USA). The Annexin V-Alexa 488 was from Invitrogen (Waltham, MA, USA), while the propidium iodide was from Santa-Cruz Biotechnology. The Hoechst 33342 was purchased from Sigma (Burlington, MA, USA). The DiL and DiO dyes were from Vybrant Cell-Labeling Solutions, Invitrogen.

### 2.2. Nanobody Production and Purification

Preparative quantities of the selected nanobodies were produced as His-tag fusion proteins in *E. coli* BL21DE3. The selected VHH sequences were preliminary cloned into expression vector pET32b+. Expression of the VHH fragments was promoted via autoinduction in ZYM-5052 medium. The cells were incubated for about two days at RT with persistent stirring and then precipitated by means of centrifugation at 6000× *g* for 8 min at 4 °C. The pellets were lysed in buffer (50 mM Tris, pH 8.5, 0.2 mg/mL lysozyme, 8 mM MgCl_2_, 0.1% Triton X-100, 10 mg/mL DNase, 10 mg/mL RNAse A) and then precipitated at 17,000× *g* for 20 min at 4 °C. The extracted proteins were purified using Ni-NTA IMAC resin (Bio-Rad). The supernatants were incubated with resin for 1 h at 4 °C. The sorbent was washed and the nanobodies were eluted with 0.5 M imidazole. The samples then were subjected to gel-filtration in a HiPrep Sephacryl S200HR column (Cytiva) into phosphate-buffered saline. The purity of the eluted nanobodies was assessed via Coomassie brilliant blue-stained 15% SDS-PAGE.

### 2.3. Biotinylated Nanobody and Streptabody Production

The pET32b+ expression vectors encoding the selected VHH sequences were modified to enable the addition of the biotin acceptor domain BAD (GGLNDIFEAQKIEWH) to the nanobody via the IgA hinge linker. The biotin acceptor domain BAD (GGLNDIFEAQKIEWH) and IgA hinge oligonucleotides sequences were artificially synthesized, annealed to each other and then cloned into pET32b+ expression vectors encoding the selected VHH sequences. The BAD–hinge construct contained restriction sites for BamHI and SalI, respectively, for the forward and reverse primers: BAD dir 3′agagagGGATCCGTCTCCGTCTACTCCGCCAACTCCGTCTCCGTCTACTCCGCCAGCTAGCGGAGGCCTGAACGATATTTTCGAAGCTCAG-5′ and BAD rev 3′agagagGTCGACTCAGTGATGATGGTGATGATGGTATTTGTATTTGCTCGAGCCGTGCCATTCGATTTTCTGAGCTTCGAAAATATCGTTCAGGCCTC-5′. The *E.coli* BL21DE3 was transformed with the BirA plasmid taken out from the *E. coli* AVB101. The cells carrying BirA were then transformed with pET32b + VHH-BAD. Expression of BAD nanobodies and BirA was autoinduced overnight, as previously described, with the addition of 0.3 mM biotin. Biotinylated nanobodies were purified using an Ni-NTA IMAC column and then via a UNO S1 cation exchange column in 50 mM PBS, pH 7.4. The biotinylated nanobodies were incubated with commercial streptavidin in vitro at RT at a molar ratio of 6:1, respectively, for 30 min to form tetrameric streptabody molecules. The obtained biotinylated nanobodies and streptabodies were assessed using Coomassie brilliant blue-stained 15% SDS-PAGE and Western blot analysis with streptavidin–HRP (1:1000).

### 2.4. Cell Culture

The U937 cells were propagated in RPMI1640, while the HEK-293, HEK-293T and MCF7 cell lines were cultured in DMEM/F12 media, under conditions of 37 °C and 5% CO_2_. The culture media contained 10% fetal bovine serum (FBS; Gibco), penicillin-streptomycin, L-glutamine, and non-essential amino acids (Gibco). The B6H12.2 hybridoma cell line HB9771 was cultured in IMDM (ATCC) with 2% FBS for monoclonal antibody production.

### 2.5. Cell Survival Assay

The U937 suspension cells were inoculated into a 24-well plate at a concentration of 10^5^ cells per well. The biotinylated B6H12.2 antibodies and biotinylated VHH fragments (67 nmol) were immobilized onto streptavidin-conjugated magnetic beads (33.5 µL) for 1 h at room temperature and then introduced to the cells. The apoptotic cells were stained with annexin-V and propidium iodide at 6 and 8 h of co-incubation, followed by detection via flow cytometry.

### 2.6. Competitive ELISA Assay

CD47 was dissolved in PBS and sorbed to a 96-well plate at a concentration of 50 ng per well and left to incubate overnight at 4 °C. The following morning, the wells were rinsed with 0.05% PBST and then blocked using 2% BSA/PBS (*w/v*) at 37 °C for 1 h. Subsequently, the wells were washed and incubated with either B6H12.2 antibodies or VHH fragments (native, biotinylated, or streptabody) at varying concentrations (12.5–2000 nM) for 1 h at 37 °C. After thorough washing, SIRPa was applied at a concentration of 50 ng per well and left for 1 h at 25 °C. Any SIRPa bound to the wells was detected using anti-SIRPa antibodies (1:800 dilution) and secondary anti-species antibodies conjugated with horseradish peroxidase (HRP). Incubation with each type of antibody was conducted at 37 °C for 1 h. Tetramethylbenzidine (TMB; Sigma) served as the substrate, and the absorbance was evaluated at 450 nm. All the experiments were performed in triplicate.

### 2.7. Phagocytosis Assay

The phagocytosis assay was performed using macrophages differentiated from PBMC. The blood was collected from a healthy donor, mixed in PBS, and layered onto Ficoll 1.077. The mixture underwent centrifugation at 400× *g* for 30 min at room temperature. Following centrifugation, the PBMC layer was collected and washed twice with PBS. The PBMCs were then cultivated in RPMI1640 medium supplemented with 10% FBS and 100 ng/mL macrophage colony-stimulating factor (M-CSF) for approximately one week at 37 °C and 5% CO_2_. The culture medium was refreshed every three days. After one week, the macrophages were harvested and stained with the red fluorescent dye, DiL (5 µL of dye per 10^6^ cells). The MCF7 and U937 cells were detached with trypsin and labeled with the green fluorescent dye, DiO, following the same protocol. The stained MCF7 or U937 cells were combined with macrophages and reseeded into 12-well plates at a density of 2 × 10^5^ cells and 10^5^ cells per well, respectively. The cocultures were incubated either with 400 nM of the VHH antibody (native, biotinylated, and streptabody forms) or with the B6H12.2 monoclonal antibody (commercial and hybridoma-purified forms) for three days. Finally, the cells were analyzed using flow cytometry.

### 2.8. Lentiviral Vector Retargeting

The pLCMV–tagRFP lentiviral vector expression plasmid was constructed previously. The psPAX2 and pMD2.G were a gift from Didier Trono (Addgene plasmid # 12260 and 12259). The pCG-Hc∆18-AA was a gift from Jakob Reiser (Addgene plasmid # 86559). The pCG-4AHc∆24-AA was constructed from pCG-Hc∆18-AA via PCR amplification with the primers Hd24-plasmid 4A BamHI dir (agagagggatccagggtgcaagatcatccacaatggccgctgcagccaaccgggagcacctgatg) and Hd24-plasmid rev (ctgatgtctatttcacactagtacaaac) and subsequent cloning with SpeI and BamHI restriction sites. The pCG-VSVTM-CT was constructed from pCG-Hc∆18-AA by subcloning with same restriction enzymes from the product of the overlap-extension PCR performed on the pET32b + VHH-BAD with the primers IgA Hinge BglII dir (agagagagatctctagaagagaagagagagagagGGATCCGTCTCCGTCTACTCCGCCAACTCCGTCTCCGTCTAC) and Iga hinge rev (gaggcaatagagcttttTGGCGGAGTAGACGGAGACGGAGTTG) and pMD2.G with the primers VSV-CT dir (GCCAaaaagctctattgcctc) and VSV-CT spe rev (tctctctactagtttactttccaagtcggttcatc). The 47VHH1H4 was subsequently subcloned in all the constructs within XbaI and BamHI restriction sites. All the transfections were performed with polyethyleneimine 25k (Polysciences) according to the recommendations provided in [25]. The ratio of the plasmids for the syncytia-formation test was 5:7:1 (pLCMV-tagRFP:pMD2-Fd30:H-protein-coding plasmid) or 5:7:1:1 (pLCMV-tagRFP:pMD2-Fd30:pCG-Hc∆18-AA:pCG-47VHH1H4-VSVTM-CT), while for the lentivirus vector packaging, the ratios were 8:8:7:1 (pLCMV-tagRFP:psPAX2:pMD2-Fd30:H-protein-coding plasmid) and 8:8:7:1:1. Moreover, 24 h after transfection, the media were changed to DMEM/F12 supplemented with PeproGrow serum replacement solution (Peprotech), and the virus-containing media were later collected after 48h of incubation, filtered through 0.45 um syringe filters and used for the transduction of HEK-293 cells via the standard spinoculation procedure with added polybrene (Sigma, 8 ug/mL). The cells were examined 72 h post-transduction via fluorescent microscope, and the viral titers were determined using flow cytometry.

### 2.9. Murine Xenograft Model

The U937 cells were administered subcutaneously at a dose of 1 × 10^6^ cells into the hind regions of 4- to 6-month-old male Balb/c nude mice (day 0). Each group, comprising five mice (*n* = 5), received treatment on the third, eighth, and fourteenth days. The treatments entailed intravenous retroorbital injections of either the B6H12.2 antibody, strepto-47VHH1H4, or anti-M13 antibody (serving as the isotype control group). Each injection comprised 100 µg of the relevant substance, prepared in 100 µL of sterile-filtered PBS. At the commencement of treatment, none of the mice presented visible tumors. The sizes of the xenografts were evaluated on days 3, 7, 8, 9, 11, 14, 17, and 22 via measuring with a caliper. The two longest perpendicular axes in the x/y plane of each xenograft were determined with an accuracy of 0.5 mm. The xenograft tumor volumes were calculated according to the formula: V = L × W^2^/2 [26].

### 2.10. Statistical Analyses

The statistical analysis was performed using GraphPad Prism 9.

## 3. Results

### 3.1. Isolation of CD47-Specific VHH Antibodies from the Library

**Figure 1:**
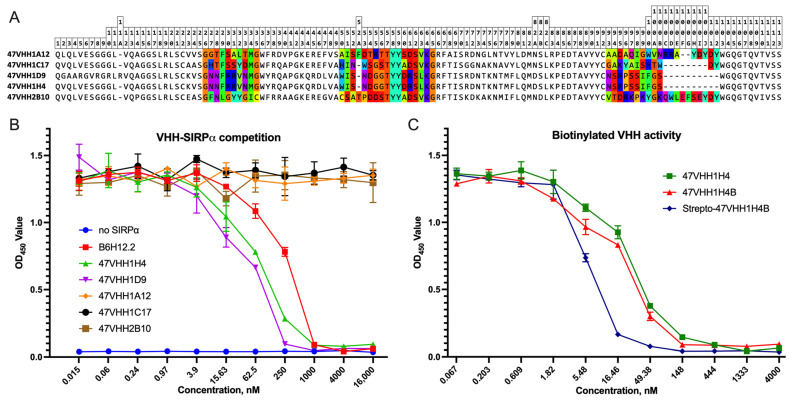
(**A**)—Aligned amino acid sequences of selected VHH clones with Kabat numbering (top). CDR regions are colored. (**B**)—The relationship between the nanobody concentration used in the competitive ELISA and the amount of SIRPa retained in its complex with CD47. IC_50_: B6H12.2 = 233.1 (194.5–313.8), 47VHH1H4 = 58.86 (48.34–85.06). (**C**)—Competitive ELISA results. IC_50_: 47VHH1H4 = 23.89 (20.64—27.15), 47VHH1H4B = 18.26 (14.76–21.76), strepto-47VHH1H4B = 5.901 (5.541–6.261).

We constructed a VHH phage display library using the peripheral blood B-cells from an alpaca that was immunized with an extracellular fragment of CD47. From the resultant phages, we selected several VHH clones for sequencing. The sequence analysis revealed that these selected fragments varied in their CDRs and were members of the VHH subfamily (Figure 1A). ELISA screening of the periplasmic extracts showed that only five clones, specifically 1H4, 1A12, 1C17, 1D9, and 2B10, exhibited specific binding to immobilized CD47, as indicated by the high signal intensity. These clones were subsequently chosen for more in-depth analysis.

### 3.2. Affinity Assessment in the Competitive Replacement of the SIRPa Ligand

To assess the binding efficiency of the chosen nanobodies, we conducted a competitive sandwich ELISA. We produced preparative amounts of nanobodies as His-tag fusion proteins in *E. coli* BL21DE3. These were then purified using metal-chelate chromatography followed by gel-filtration. The purity of the nanobodies was confirmed via SDS-PAGE (Figure S1), which indicated a molecular weight of roughly 17 kDa. We used human SIRPα-Fc as CD47’s natural ligand, with the CD47 monoclonal antibody B6H12.2 serving as a benchmark. Based on the competitive immunoassay outcomes, the VHH clone 47VHH1D9 exhibited the greatest potency (Figure 1B). Following this in terms of the blocking potency were the VHH clone 47VHH1H4 and B6H12.2. Meanwhile, the VHH clones 47VHH1A12, 47VHH1C17, and 47VHH2B10 showed no capacity to disrupt the CD47–SIRPα interaction.

We subsequently measured the surface plasmon resonance of each pair of the selected nanobodies and a recombinant CD47 anchored to a CM5 chip using the Biacore T200 system. The results revealed that 47VHH1H4 exhibited a binding strength of 30 nM, signifying a notably high affinity. This nanobody’s binding capability surpassed that of the commercial murine antibody B6H12.2, which had a Kd value of roughly 50 nM (this includes the avidity effects). The other VHH clones displayed binding strengths in the micromolar range: 47VHH1D9 had a Kd of 400 nM, 47VHH1C17 showed a Kd of 2 μM, while both 47VHH1A12 and 47VHH2B10 were around 30 μM.

### 3.3. Biotinylated Antibody Production and Assessment

One recognized method to enhance the avidity of antibody fragments is through tetramerization on streptavidin, leading to the formation of a “streptabody” molecule. We modified the expression vector encoding 47VHH1H4 to allow for the addition of the biotin acceptor domain (BAD)—GGLNDIFEAQKIEWH—to the VHH antibody using an IgA hinge linker. The resulting biotinylated 47VHH1H4 fragment, termed 47VHH1H4B, was purified using an Ni-NTA column. It was then attached to streptavidin in vitro, forming the tetrameric streptabody molecule called strepto-47VHH1H4B. The purity of the 47VHH1H4B and the changes in its molecular weight were verified through SDS-PAGE and Western blot using streptavidin–HRP staining (Figure S2). The observed molecular weight of the 47VHH1H4B was approximately 25 kDa.

The affinity of the generated 47VHH1H4B was assessed using a competitive ELISA. This analysis revealed that the 47VHH1H4 streptabody molecule had superior affinity, as shown in Figure 1C.

Using the Biacore T200, the dissociation constant of the 47VHH1H4B was determined to be 3 nM. This represents a tenfold enhancement of the binding strength of the biotinylated antibody compared to its native counterpart.

### 3.4. Survival Assay

We evaluated the activity of the 47VHH1H4B anti-CD47 nanobody using an apoptosis assay, specifically targeting the U937 monocytic lymphoma cell line known for its pronounced CD47 surface expression. Numerous studies have demonstrated that U937 cells are highly sensitive to CD47 blockade, which can sometimes result in direct apoptosis [27]. Leveraging this insight, we investigated whether our anti-CD47 nanobody could similarly compromise U937 cell survival.

B6H12.2-dependent apoptosis is known to be triggered when antibodies are anchored to a surface [28]. For our study, we employed magnetic microspheres coupled with biotinylated antibodies. This design ensures interaction between the cells and antibodies throughout the U937 culture, which is in a suspension. Equal molar quantities of the immobilized and soluble forms of 47VHH1H4B and biotinylated B6H12.2 were introduced to the U937 cells. We employed camptothecin, a known apoptosis inducer, as our positive control. Furthermore, biotinylated anti-CD45 antibodies were utilized to ascertain that the reduced cell survival was specifically due to CD47 blockade.

**Figure 2:**
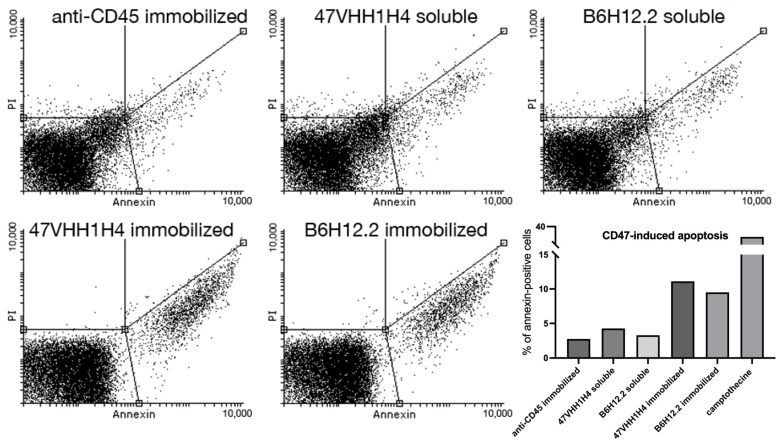
Assessment of apoptotic and necrotic cell percentages eight hours after U937 treatment with 47VHH1H4B, biotinylated B6H12.2, and biotinylated anti-CD45 antibody.

The assay demonstrated that the samples treated with immobilized B6H12.2 and 47VHH1H4 exhibited apoptotic cell percentages of 9.5% and 11%, respectively (Figure 2). In contrast, the camptothecin-treated cells exhibited a 37% rate. This discrepancy could be attributed to either the insufficient antibody concentrations or the experimental setup. Nevertheless, an increase in apoptotic cells was observed six hours post-treatment, and this surge continued until the eight-hour mark for both the soluble and immobilized forms of B6H12.2 and 47VHH1H4B antibodies. This confirms the specificity of our 47VHH1H4B nanobody and the known fact that immobilized CD47-blocking antibodies induce apoptosis in this cell culture. It suggests that B6H12.2 and 47VHH1H4B may target epitopes that either overlap or are in close proximity. Furthermore, the visible superior efficiency of 47VHH1H4B relative to B6H12.2 indicates its heightened potency.

### 3.5. Phagocytosis Assay

Next, we investigated whether blocking CD47 on cancer cell membranes using nanobodies could enhance their phagocytosis by macrophages. We selected the U937 and MCF7 cancer cell lines for this experiment due to their pronounced CD47 expression levels. M1 macrophages, which are known to exhibit phagocytic activity against cancer cells, were cultivated from the blood of a healthy donor. This was achieved by treating the peripheral blood mononuclear cells (PBMCs) with MCSF. These macrophages were then introduced to the previously plated U937 and MCF7 cells. Simultaneously, these cells were exposed to the native 47VHH1H4, its biotinylated form (47VHH1H4B), or the streptabody conjugate (strepto-47VHH1H4). For comparison, we also treated cells with commercially available B6H12.2 (sc) and B6H12.2 purified from hybridoma supernatants (nat). To trace the phagocytosis visually, we stained the macrophages with the fluorescent membrane dye DiO, while the cancer cells were labeled with the vital dye DiL.

**Figure 3:**
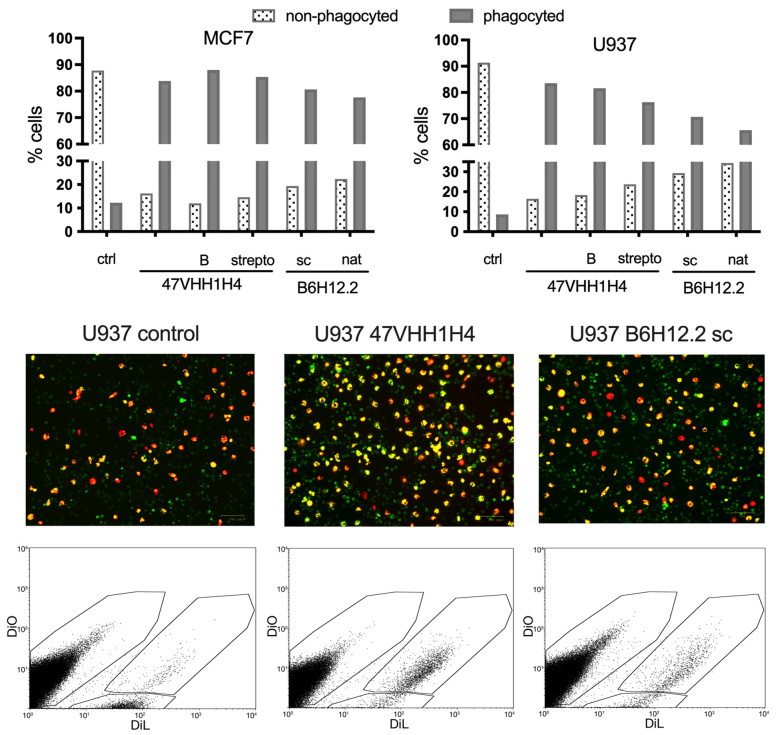
Proportion of phagocytosed vs. non-phagocytosed MCF7 and U937 cells following 72 h of co-incubation with M1 macrophages. Values depict the ratio of phagocytosed or non-phagocytosed cancer cells relative to the total cancer cells. The 47VHH1H4 group includes treatments with native 47VHH1H4, biotinylated 47VHH1H4B (B), and strepto-47VHH1H4 (strepto). The B6H12.2 group refers to treatment with commercial antibodies from Santa-Cruz (sc) and in-house purified B6H12.2 from hybridoma supernatants (nat). Red – DiL-stained macrophages, Green – DiO-stained U937 cells.

Treatment with the anti-CD47 nanobody fragments resulted in a notable increase in the number of phagocytosed MCF7 and U937 cancer cells (Figure 3). The proportion of phagocytosed MCF7 cells was fairly consistent across the groups: 83% for the native 47VHH1H4, 87% for the biotinylated 47VHH1H4B, and 85% for the streptabody. For the U937 cells, the native 47VHH1H4 was the most effective, leading to 83% phagocytosis, while the 47VHH1H4B and streptabody treatments resulted in 81% and 76% phagocytosed cells, respectively. In the best-case scenarios, B6H12.2 led to 80% phagocytosis of the MCF7 cells and 70% of the U937. Based on these results, 47VHH1H4 effectively blocks the CD47–SIRPa interaction. There was no significant difference between the modified and native 47VHH1H4 treatment groups. However, 47VHH1H4 generally exhibited a higher efficiency in stimulating phagocytosis compared to the commercial B6H12.2.

### 3.6. Retargeting of Lentiviral Vectors with 47VHH1H4

Besides enhancing phagocytosis via inhibiting the interaction between CD47 and SIRPa, anti-CD47 nanobodies might offer a targeted approach to deliver transgenes to tumor cells expressing CD47. For this application, we employed lentiviral vectors pseudotyped with the receptor-blinded measles virus glycoprotein H, coupled with a targeting nanobody.

To evaluate the efficacy of the 47VHH1H4 nanobody, it was integrated into the packaging plasmids pCG-Hd18AA-XB [29] and pCG-4AHd24AA-XB. Both of these constructs contain the mutated H protein sequence with either 18 or 24 N-terminal amino acids excluded. 47VHH1H4 was also incorporated into the pCG-VSVTM-CT construct that houses the sequence for the transmembrane and cytoplasmic domains of the vesicular stomatitis virus G protein, resulting in the formation of the 47VHH1H4 pseudoreceptor [30].

An initial evaluation of 47VHH1H4’s capability to trigger fusion between viral and cellular membranes using the F protein was conducted through a syncytia formation test. This involved cotransfecting the HEK-293T cells with several plasmids: pLCMV-tagRFP (encoding red fluorescent protein), pMD2-Fd30 (encoding the measles virus’s F protein), and one of the plasmids encoding the receptor-neutralized H protein (H-AA) attached to 47VHH1H4. Alternatively, plasmids encoding H-AA and the 47VHH1H4 pseudoreceptor were used.

**Figure 4:**
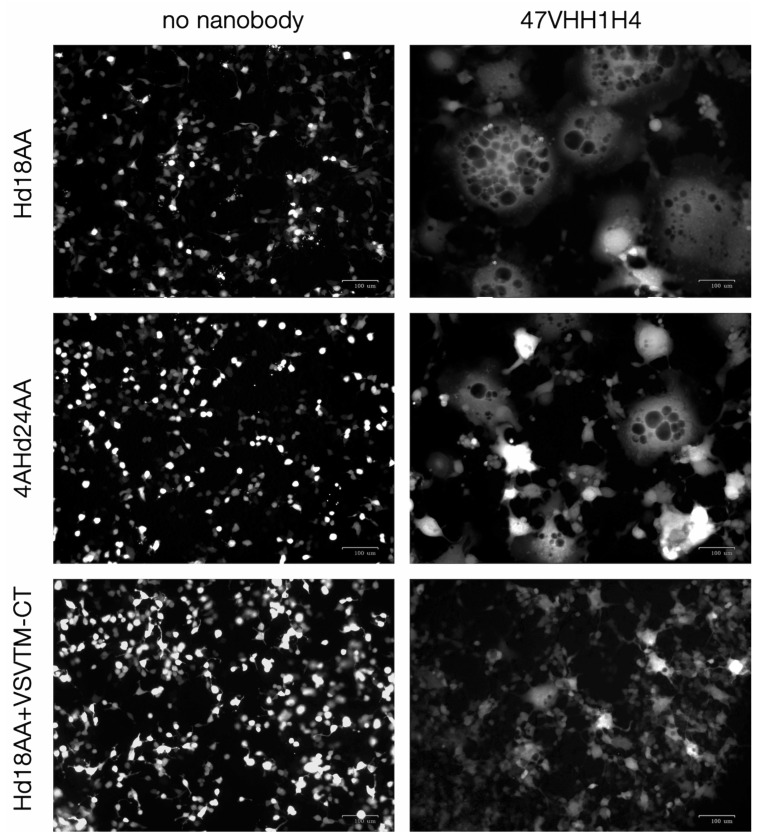
Syncytia formation after transfection with plasmids coding 47VHH1H4-retargeted measles glycoproteins. Left column—control samples, transfected with pCG-Hd18AA, pCG-4AHd24AA-XB, and pCG-VSVTM-CT + pCG-Hd18AA-XB. Right column—samples transfected with pCG-Hd18AA-47VHH1H4, pCG-4AHd24AA-47VHH1H4 and pCG-47VHH1H4-VSVTM-CT + pCG-Hd18AA-XB.

Figure 4 illustrates that employing the constructs pCG-Hd18AA-47VHH1H4 and pCG-4AHd24AA-47VHH1H4 resulted in the pronounced formation of fluorescent syncytia. In contrast, cotransfection with the control plasmids pCG-Hd18AA-XB and pCG-4AHd24AA-XB yielded negligible results. Transfection combining the plasmids pCG-Hd18AA-XB and pCG-47VHH1H4-VSVTM-CT produced only isolated, small syncytia formations.

Subsequently, lentiviral vector batches were produced by cotransfecting with these plasmids and adding the psPAX2 packaging plasmid to the mix. The fully functional plasmid pCG-4AHd24 served as a positive control. We used supernatants containing lentivectors to transduce HEK-293 cells. The subsequent viral titer was then quantified using flow cytometry, as outlined in Table 1.

Our experiments revealed that lentiviral particles redirected toward CD47 with 47VHH1H4 possess limited infectious capabilities. Among the three constructs evaluated, only the receptor-blinded H protein combined with the 47VHH1H4 pseudoreceptor resulted in detectable transduced cells. This suggests that the binding site of 47VHH1H4 on CD47 may not be ideal for retargeting purposes.

### 3.7. Tumor Growth Suppression

The ability of CD47-blocking agents to induce apoptosis of CD47-hyperexpressing cancer cells may allow the suppression of tumor growth in a tumor xenotransplant animal model [31]. To test whether the 47VHH1H4 possesses such activity, groups of tumor-bearing Balb/c nude mice were treated either with intravenous injections of the B6H12.2 antibody or strepto-47VHH1H4B. The tetrameric form was chosen for its estimated longer clearance compared to the monomeric 47VHH1H4. The control group received the anti-M13 antibody.

**Figure 5:**
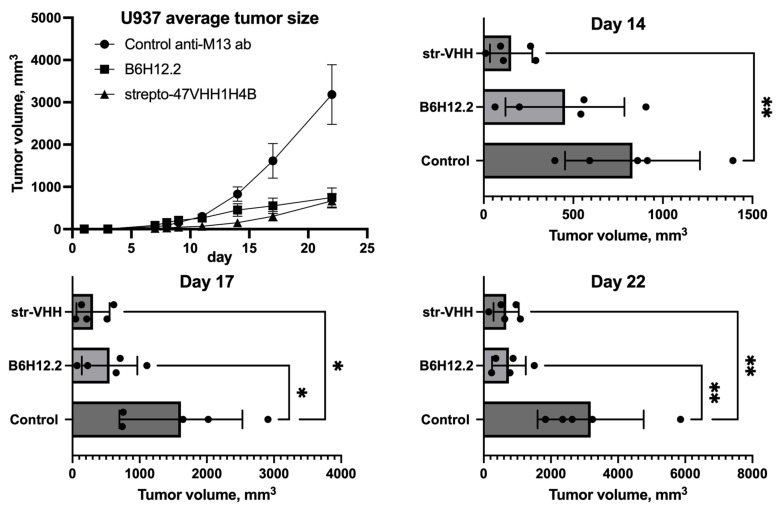
U937 tumor xenotransplant growth dynamic in Balb/c nude mice treated with B6H12.2, strepto-47VHH1H4B or control anti-M13 antibody. *—*p* < 0.033, **—*p* < 0.0021.

The strepto-47VHH1H4B group began to deviate from the control group in terms of the average tumor volume on day 8, and on day 14, this deviation passed the significance threshold (Figure 5). Starting from day 17, the B6H12.2 group demonstrated a significantly lower average tumor volume than the control group, and at the endpoint (day 22), both experimental groups had median tumor volumes three times lower than the control group. This result indicates that 47VHH1H4 is able to efficiently suppress tumor growth in vivo and that strepto-47VHH1H4B is at least as efficient as B6H12.2 in this setting.

## 4. Discussion

The CD47 receptor is frequently overexpressed in tumor cells of various origins. High levels of CD47 form a more permissive microenvironment that allows tumor evasion from innate immunity attack [32]. CD47 blockade may function as an independent antitumor therapy or a complementary component in complex therapeutic regimens [33]. The use of new therapeutic approaches, including CAR-T-lymphocytes, viral oncolytics, and dendritic cell vaccines, may require a CD47-targeted treatment component with distinctive physicochemical properties to achieve maximum efficacy. In this case, the use of full-length antibodies may not be optimal, and nanobodies, owing to their compact size, superior tissue permeability, and stability, may prove more suitable [34].

The results obtained by us during the comparative testing of the 47VHH1H4 nanobody indicate that it has full ability to functionally block CD47—it effectively interrupts the interaction of CD47 and its ligand SIRPα. This disruption enables macrophages to initiate ex vivo phagocytosis of tumor cells. Moreover, CD47 ligation using an immobilized 47VHH1H4 leads to apoptosis of U937 tumor cells characterized by CD47 overexpression. In these tests, 47VHH1H4 demonstrated slightly better efficiency than the full-length blocking antibody B6H12.2, which aligns with the binding affinity data concerning 47VHH1H4 and CD47, where the dissociation constant (Kd) of this interaction is in the lower nanomolar range. This evidence collectively suggests the potential of 47VHH1H4 as a potent CD47-blocking agent for cancer immunotherapy.

CD47, when overexpressed in tumor cells, can serve as a potential target for the directed delivery of expression cassettes with genetically encoded therapeutic agents [35] or for the targeting of viral oncolytics [36]. In our study, we evaluated the 47VHH1H4 nanobody for this purpose. While it did activate the measles virus F protein to induce cell membrane fusion, the infectious titer of the retargeted lentiviral vectors was substantially lower (by over three orders of magnitude) compared to the non-retargeted version. This suggests that the specific region of the protein recognized by 47VHH1H4 might not be ideally situated for the proper alignment of cellular and viral membranes before fusion, as most targeting moieties that were successfully used for lentivector retargeting caused less substantial drops in the viral titer [37,38].

Beyond their in situ expression, CD47 blockers may also serve as essential components in multifunctional protein therapeutics [39], antibody-drug conjugates [40], or other molecular constructs [41]. To evaluate the potential of 47VHH1H4 in this role, we created a prototype polyvalent complex. Through biotinylation in vivo using biotin ligase, we produced a 47VHH1H4B variant bearing single C-terminal biotin and assembled a tetravalent streptabody based on it. This molecule is anticipated to have an extended half-life due to its size exceeding the glomerular filtration barrier, and the presence of four nanobodies in its composition should enhance its affinity for CD47 owing to avidity [42]. Furthermore, the dissociation constant (Kd) measurements for 47VHH1H4 and 47VHH1H4B revealed that BAD-containing forms had slightly higher affinities for CD47 regardless of their biotinylation status. This can potentially be attributed to its more efficient transportation to the periplasm and, consequently, the enhanced stability of the secondary structure due to the complete formation of disulfide bonds under nonreducing conditions [43]. In the ELISA assays, we demonstrated that strepto-47VHH1H4B exhibited activity at lower molar concentrations compared to 47VHH1H4, which was further confirmed by the phagocytosis assay results. In vivo experiments revealed that strepto-47VHH1H4B slightly outperformed B6H12.2 in terms of efficacy. Since the introduction of therapy was carried out with an interval of 5–6 days, we infer that strepto-47VHH1H4B possesses an extended half-life. In mice with a BALB/c background, SIRPα exhibits a relatively low affinity for human CD47 (~0.3 µM [44]). It is plausible that both strepto-47VHH1H4B and B6H12.2 effectively outcompeted SIRPα in binding to the tumor receptor, which may explain why the difference in affinity between strepto-47VHH1H4B and B6H12.2 did not significantly alter the tumor growth dynamics. It could be speculated that this result could be substantially improved in syngeneic tumor models or in NOD background mice [45].

The construction of streptabodies facilitates the most rapid assembly of tetravalent complexes from nanobodies that have undergone in vivo biotinylation. This method enables the evaluation of nanobodies’ antitumor effects in animal models and potentially enhances their efficacy via the avidity effect. There are, however, inherent limitations associated with streptabodies. The tetrameric structure of streptavidin is immunogenic, and the biotin present in blood plasma may displace biotinylated nanobodies over time, potentially diminishing their therapeutic impact. Although there have been attempts to engineer streptavidin variants with reduced immunogenicity [46], and while several studies indicate that streptavidin-conjugated therapeutics maintain considerable stability in the presence of physiological biotin concentrations [47,48], alternative strategies for creating nanobody oligomers may offer superior benefits. Specifically, the employment of bioconjugation techniques involving recombinant sortase A and bioorthogonal agents for click chemistry [49] may yield nanobody tetramers [50] that are more compact, stable, and exhibit reduced immunogenicity.

Although our attempt to retarget lentiviral vectors using 47VHH1H4 in conjunction with measles glycoproteins did not yield positive results, the generated constructs, especially the 47VHH1H4-based pseudoreceptor, present opportunities for further experimentation in combination with other viral glycoproteins. This approach is supported by recent advancements in lentiviral vector retargeting, particularly by the successful employment of a receptor-blinded variant of VSV-G combined with a targeting pseudoreceptor to achieve effective retargeting of lentivectors [51].

To date, therapeutic agents based on nanobodies that target the CD47–SIRPα signaling axis are being used as independent therapeutic agents, components of immunotherapy drugs, or auxiliary components for complex therapy. Nevertheless, the potential applications of anti-CD47 nanobodies extend beyond these uses. In particular, they could serve as payloads of recombinant viral oncolytics. This suggests that diverse variants of such nanobodies, which functionally block CD47 by binding to different epitopes, might be required for a range of applications. These nanobodies could serve as invaluable tools in cancer research, facilitating a deeper understanding of the CD47–SIRPα signaling axis and its role in tumor progression and immune evasion.

## 5. Conclusions

A novel specific anti-CD47 nanobody from an immunized alpaca was engineered, exhibiting strong potency in disrupting the CD47–SIRPα interaction, a major axis in cancer immunotherapy. Compared to the widely recognized monoclonal antibody B6H12.2, our nanobody displayed superior affinity, substantiating its therapeutic potential. Through various modifications, including biotinylation and the formation of streptabodies, we achieved enhancements in the nanobody’s affinity, further widening its range of possible therapeutic applications. The anti-CD47 nanobody facilitated significant phagocytosis of the U937 and MCF7 tumor cell lines while also inducing apoptosis in the U937 cells both ex vivo and in vivo in mouse xenotransplants. These findings validate the robust affinity and functional activity of the nanobody and pave the way for its exploration in more extensive in vivo studies. As the realm of cancer immunotherapy grows, the inclusion of this nanobody, either as a standalone agent or in combination therapies, could be instrumental in advancing treatment options for patients.

## Figures and Tables

**Table 1:** Infectious titers of lentiviral vectors retargeted to the CD47 receptor.

Pseudotyping Construct	Syncytia on Packaging Cells	Infectious Titer
pCG-4AHd24	+	~7 × 10^4^
pCG-Hd18AA-XB	−	<10^2^
pCG-4AHd24AA-XB	−	<10^2^
pCG-Hd18AA-47VHH1H4	++	<10^2^
pCG-4AHd24AA-47VHH1H4	+	<10^2^
pCG-47VHH1H4-VSVTM-CT + pCG-Hd18AA-XB	+/−	3 × 10^2^

## Data Availability

The data presented in this study are available in the manuscript.

## Supplementary Materials

The following supporting information can be downloaded at: https://www.mdpi.com/article/10.3390/antib13010002/s1, Figure S1: Coumassie stain of SDS-PAGE of purified nanobodies; Figure S2: Western blot of 47VHH1H4B developed with streptavidin-HRP (lane 1) and Coumassie stain of SDS-PAGE of 47VHH1H4B (lane 2).
