# Supplementary material for: A Novel Anti-CD47 Nanobody Tetramer for Cancer Therapy
Source: Antibodies (Basel). 2024 Jan 2;13(1):2. doi: 10.3390/antib13010002 (PMC10801496; doi:10.3390/antib13010002)
Supplement: Supplementary file 1 [file antibodies-13-00002-s001.zip › antibodies-2721902-supplementary.pdf]

# Supplementary Information

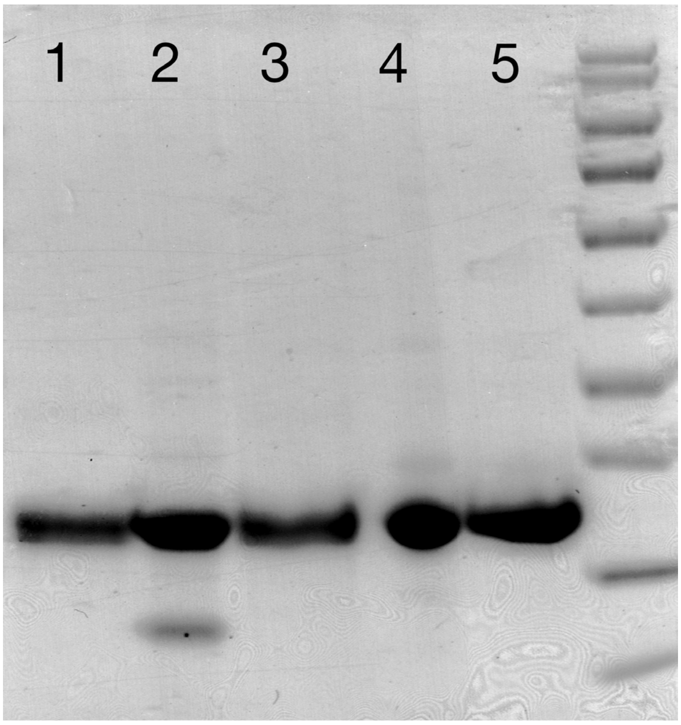


**Figure S1.** Coumassie stain of SDS-PAGE of purified nanobodies. 1 – 47VHH1A12, 2 – 47VHH1C17, 3 – 47VHH2B10, 4 – 47VHH1D9, 5 – 47VHH1H4

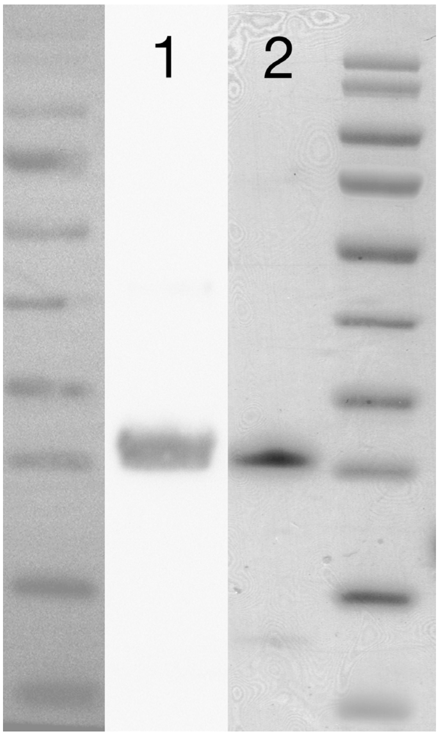


**Figure S2.** Western blot of 47VHH1H4B developed with streptavidin-HRP (lane 1) and Coumassie stain of SDS-PAGE of 47VHH1H4B (lane 2)

# Supplementary Methods

*Alpaca immunization and VHH nucleotide sequences acquisition*

About 700 µg of recombinant human extracellular domain of CD47 was dissolved in 500 µl of PBS and then was mixed with an equivalent volume of Freund's complete adjuvant (Pierce, USA). The mixture was injected into the hind right leg of Alpaca (*Vicugna pacos*). Two additional boosts were performed with intervals of 3 weeks. Five days after last boost, 120 ml of venous blood was collected from the external jugular vein. The blood was then separated in ficoll gradient and PBMC fraction was isolated. The total RNA was extracted from PBMC using tri-reagent, and then obtained RNA was converted into cDNA by reverse transcription. The cDNAs for the VHH fragments were amplified by standard PCR reaction and then by nested PCR using set of primers contained restriction sites: direct AlpVHH-F1X 5′ -TCTAGAGAGATCGCCGGCCAGKTGCAGCTCGTGGAGTCNGGNGG-3′ and reverse AlpVHH-R1B 5′-GGATCCGATCACTAGTGGGGTCTTCGCTGTGGTGCG-3′ or AlpVHH-R2B 5′-GGATCCGATCACTAGTTTGTGGTTTTGGTGTCTTGGG-3′. Next, the obtained PCR fragments were cloned into pHEN2-XB phagemid vector and electroporated into electro-competent *E. coli* TG-1. Bacterial library was converted to phage library after infection with helper phage M13K07 (20 phages per 1 bacteria). The phage library was purified and concentrated by precipitation with PEG/NaCl (20% PEG 6000, 2.5M NaCl).

*Library enrichment*

Selection of specific anti-CD47 nanobodies was performed using three consecutive rounds of biopanning procedure. The recombinant extracellular domain of CD47 receptor was used as an antigene. Immunotubes were coated with 50, 25 and 12.5 ug/ml of CD47 from first to third iteration of biopanning, respectively, and then incubated overnight at RT. Next morning, the wells were washed with PBS and blocked for 2 hours with 2% BSA/PBS at 37°C. After that, $10^{12}$-$10^{13}$ cfu of phage library dissolved in 2% BSA/PBS (w/v) were added to the tubes and incubated for 2h at RT. Unbounded phages were removed by washing 20 times with PBS containing 0.1% Tween20 (0.1% PBST, v/v). Bounded phages were eluted by 10 minutes incubation at RT with 100 mM triethylamine and then were amplified into TG-1 with consecutive adding of M13K07. The propagated phages were again precipitated by PEG/NaCl and used for the next round of panning.

*Screening for specific anti-CD47 nanobodies*

After the third round of panning, individual phage clones carrying VHH were analyzed by indirect ELISA for affinity to immobilized CD47. The CD47 antigene dissolved in PBS to 0.8-3 µg/ml was sorbed onto 96-well plate overnight at 4°C. Next day, wells were washed three times with 0,05% PBST and blocked with 3% BSA/PBS (w/v) at 37°C for 1 h. After washing with PBST, wells were incubated with $10^{12}$ cfu of randomly selected phages for 1 hour. After that, unbounded phages were removed and wells were washed three times with 0.05% PBST buffer. Bound phages were detected by anti-M13-HRP antibody. Incubation with antibodies was carried out at 37°C for 1 h. TMB (Sigma) was used as a developer and absorbance was evaluated at 450 nm. Clones showed the strongest signal were converted back to phagemid. Nucleotide sequence of nanobodies were amplified and treated with HaeIII restriction enzyme to assess diversity. Clones similar in restriction profile were pooled together and were used for the further analysis.

Selected nanobodies were produced in a periplasmic extract of *E.coli* strain HB2151 which enables to generate VHH fragments solely without fusion to PIII phage protein. Individual transformed bacterial clones were cultured in 2×YT medium with 100 µg/ml carbenicillin and 0.1% glucose until reaching log phase (OD = 0.6–0.8). Expression of nanobodies was induced by 1 mM IPTG overnight. Periplasmic proteins were extracted by osmotic shock with followed sonication and then analyzed by indirect ELISA using the protocol described previously.
